# More than a meat- or synthetic nitrogen fertiliser-substitute: a review of legume phytochemicals as drivers of ‘One Health’ via their influence on the functional diversity of soil- and gut-microbes

**DOI:** 10.3389/fpls.2024.1337653

**Published:** 2024-02-21

**Authors:** Rafael D. C. Duarte, Pietro P. M. Iannetta, Ana M. Gomes, Marta W. Vasconcelos

**Affiliations:** ^1^ Universidade Católica Portuguesa, CBQF - Centro de Biotecnologia e Química Fina – Laboratório Associado, Escola Superior de Biotecnologia, Porto, Portugal; ^2^ Ecological Sciences, James Hutton Institute, Dundee, United Kingdom

**Keywords:** legumes, phytochemicals, soil microbiome, gut microbiome, One Health, functional diversity

## Abstract

Legumes are essential to healthy agroecosystems, with a rich phytochemical content that impacts overall human and animal well-being and environmental sustainability. While these phytochemicals can have both positive and negative effects, legumes have traditionally been bred to produce genotypes with lower levels of certain plant phytochemicals, specifically those commonly termed as ‘antifeedants’ including phenolic compounds, saponins, alkaloids, tannins, and raffinose family oligosaccharides (RFOs). However, when incorporated into a balanced diet, such legume phytochemicals can offer health benefits for both humans and animals. They can positively influence the human gut microbiome by promoting the growth of beneficial bacteria, contributing to gut health, and demonstrating anti-inflammatory and antioxidant properties. Beyond their nutritional value, legume phytochemicals also play a vital role in soil health. The phytochemical containing residues from their shoots and roots usually remain in-field to positively affect soil nutrient status and microbiome diversity, so enhancing soil functions and benefiting performance and yield of following crops. This review explores the role of legume phytochemicals from a ‘one health’ perspective, examining their on soil- and gut-microbial ecology, bridging the gap between human nutrition and agroecological science.

## 1 Introduction

Legumes are a diverse group of plants grown worldwide and consumed as an essential source of food and feed for thousands of years, playing a vital role in developing agriculture and human civilization (Ferreira et al., 2021). Belonging to the *Fabaceae* family, they comprise over 19,500 species and 750 genera and account for the third largest family of flowering plants (Bruneau et al., 2013). Pulses are a subgroup of grain legumes harvested for dried grains, such as pea (*Pisum sativum* L.), lentil (*Lens culinaris* L.), chickpea (*Cicer arietinum* L.) and faba bean (*Vicia faba* L.). Cultivated by early civilizations, legumes spread worldwide providing a reliable source of balanced nutritional provision, as well as serving as key component of many traditional cuisines (Ferreira et al., 2021).

Nowadays, the importance of legumes for food security has not wavered, with the United Nations General Assembly declaring 2016 as the *International Year of the Pulses* and declaring World Pulses Day every February 10^th^ thereafter (Calles et al., 2019). Legumes are valuable sources of protein, fiber, carbohydrates and fatty acids, regularly associated as key components of a healthy and balanced diet (Geraldo et al., 2022). Also, they contain several bioactive ‘non-nutrients’ often considered as toxins or ‘antifeedants’, that lately have been associated with beneficial bioactive properties for gut microbiome function and human health (Rodríguez-Daza et al., 2021; Geraldo et al., 2022). Research suggests that flavonoids, plant sterols, oligosaccharides and others phytochemicals can act as prebiotics, promoting the growth and activity of beneficial gut bacteria (Pei et al., 2020). Scientific evidence regarding the potential of pulses phytochemicals to improve digestive health, strengthening the immune system, and potentially lowering the risk of gastrointestinal disorders through increased consumption of grain legumes is expanding rapidly (Rodríguez-Daza et al., 2021).

In contrast to pulses, forage legumes are primarily cultivated to serve as animal feed, with the entire aboveground part of the plant used as a source of nutrition for livestock. This type of legumes is also gaining popularity as a pivotal crop to realise regenerative agriculture practices, where they are recognized for their potential to enhance soil functions and qualities, serving as integrated pest management tools thus reducing the need for pesticides. Forage legumes like alfalfa (*Medicago sativa* L.) and clover (*Trifolium* L. spp.) are typically harvested at an earlier stage before they reach full flowering and seeding. This stage results in these legumes having a higher fiber content (Capstaff and Miller, 2018) and various bioactive phytochemicals, including phenolic compounds (PC), polysaccharides, and phytoestrogens (Tedeschi et al., 2021; Horvat et al., 2022). In line with their impact on human health, these compounds have been reported to have positive effects on the health of ruminants’ digestive systems and also contribute to improved soil functions (Clemensen et al., 2022; Mei et al., 2022).

Grain and forage legumes alike play a key role in providing ecosystem services. Through symbiosis with specific types of soil bacteria contained within legume root nodules, often referred collectively as rhizobia (Calles et al., 2019), inert atmospheric di-nitrogen (N) gas is ‘fixed’ into biologically useful forms in a process called biological N fixation (BNF); the primary natural source of N-cycling to support ecological systems (Iannetta et al., 2016). Compared to long-term cereal-based cropping systems, which are critical contributors for greenhouse gases (GHGs) (Dutta et al., 2022), legumes have been shown to lower emissions (Rahman et al., 2022). Additionally, incorporating the wild range of legume phytochemicals via intercropping strategies can help enhance agroecosystem resilience through improved soil fertility, biodiversity and nutrient cycling (Chamkhi et al., 2022). Consequently, legumes have been identified as pivotal tools to enhance the intended transition towards ‘healthy soil’ by 2030 (European Soil Mission) (Fendrich et al., 2023). Despite this, the production of legumes in Europe remains limited and below the desired thresholds for optimal ecological function, with grain legumes accounting for less than 3% of arable land across the EU, and are mainly used for animal feed (Notz et al., 2023).

Moreover, to cater to the demand for intensive meat production, where highly digestible diets are desired (Squire et al., 2019), commercially significant legume crops are still targeted/bred to reduce their levels of specific non-nutritional phytochemicals (commonly termed ‘antifeedants’), despite the previously mentioned potential of such chemical compounds in crop and health-protection (Geraldo et al., 2022). Breeders seek to reduce non-nutritional factors in crops for several reasons. These compounds can contribute to bitterness and astringency (Robinson et al., 2019), affect colour stability (Huang et al., 2022), hinder food processing (Poljsak et al., 2021), and, in some cases, have allergenic or toxic effects (Kumar et al., 2013). Additionally, high phenolic levels in forage crops may impact livestock health and feed palatability (Lamy et al., 2011). By minimizing specific phytochemicals, breeders were aiming to enhance the overall quality, taste, and marketability of crops while addressing potential health concerns. Notwithstanding, this approach may inadvertently undermine the overall nutritional quality of legumes and soil health-promoting effects, as discussed in the present review. Furthermore, along with the removal of such phytochemicals, other desirable traits such as yield and seed size, may be compromised (Duraiswamy et al., 2023).

Legumes therefore epitomize the causal and functional processes which exist between human-, animal-, and environmental-health within the ‘One Health’ framework – and since they also play a natural and pivotal role mediating connected soil- and climate-based chemical- and water-cycles (Lerner and Berg, 2015). As this holistic approach acknowledges that the health of these domains is intricately linked; the ‘One Health’ concept is continually to gain traction while it emphasizes the need for more-collaborative efforts across disciplines to address complex health challenges (Gibbs, 2014). By recognizing the shared health vulnerabilities across different species, ecosystems and processes, One Health advocates for integrated solutions that account for such interconnectedness and with a view to ‘global health’ - aiming to achieve optimal health outcomes for all living beings while safeguarding aspects of biodiversity (*i.e.*, including biochemical diversity), to help promote and enhance environmental sustainability and resilience (Mumford et al., 2023). Whether through enhanced gut microbiome function, or their potential to serve as favourable factors for soil microbiome diversity and soil processes to better soil-plant performance, legume phytochemicals act as crucial determining agents of ‘One Health’ in praxis.

In this review we aim to describe the qualitative profile of the highly diverse legume phytochemicals considering their biochemistry, as well as their biological and ecological significance. Scrutinising both soil and gut microbial ecology, this review critically evaluates the integration of legumes and their phytochemicals within agricultural (soil-based) systems and emphasizing their dual role as a staple in human diets and essential animal feed. Understanding legumes influence in these capacities becomes pivotal in advancing the realization of the overarching ‘One Health’ concept, underlining the interconnectedness of human, animal, and environmental health.

## 2 Legume phytochemicals, their biochemistry, and significance

**Figure 1:**
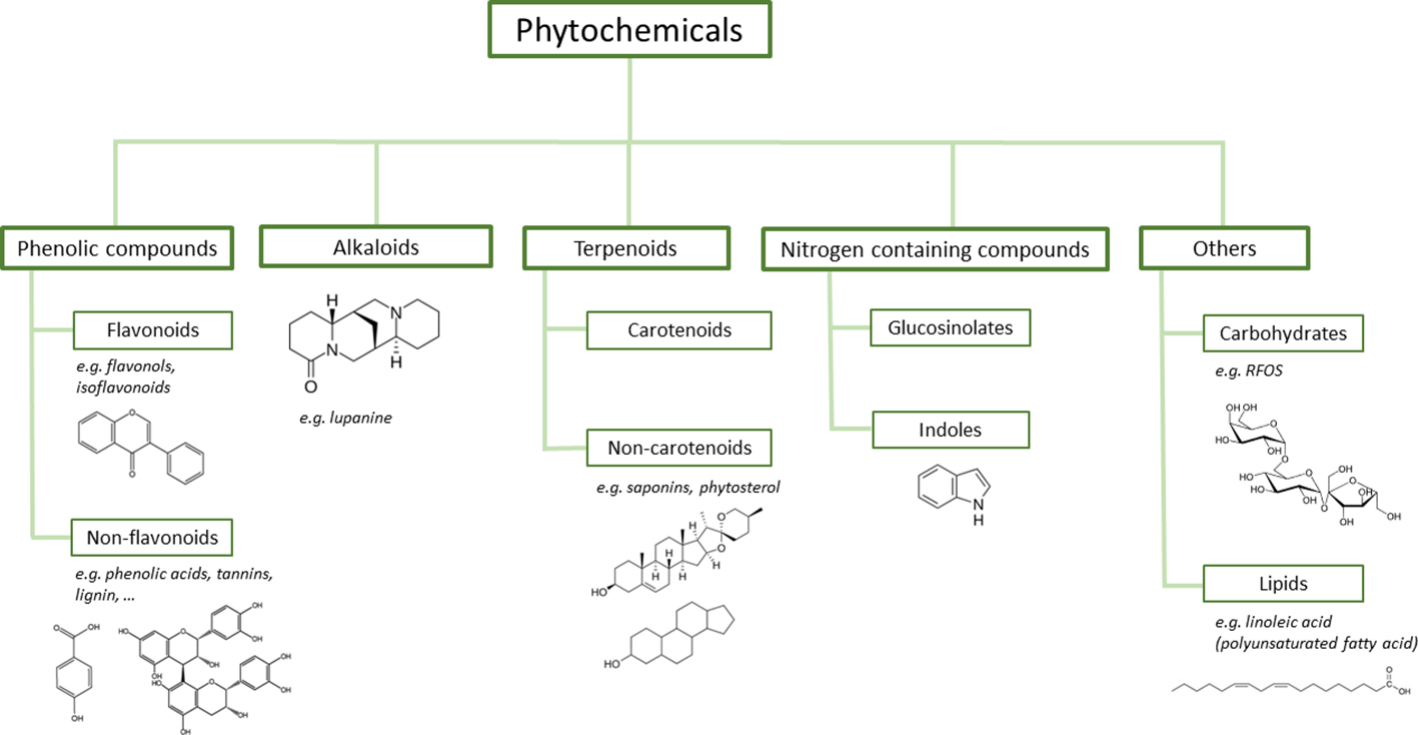
Classification and structure of commonly found legume phytochemicals.

Phytochemicals are biologically active compounds naturally produced in all plants. Notwithstanding the inference of such chemicals as ‘non-essential’, there has been an increasing interest in legumes due to their characterisation as a particularly rich source of such compounds (Tor-Roca et al., 2020). These specialised metabolites, alongside their impact on gut, soil ecology and considerable pharmaceutical importance (Eljounaidi and Lichman, 2020), can play various roles in the plants themselves. For instance, they support plant defence (Basu et al., 2022), might suppress the growth of competing plants, providing legumes with a competitive advantage (Al-Deliamy and Abdul-Ameer, 2023) and regulate certain stages of legume growth and development (Mohammed et al., 2019; Misra et al., 2023). Legumes present a wide variety of such phytochemicals and there are several ways in which these may be characterised and classified, whether by function, chemical structure, or functional groups. Main classes of such phytochemicals (**Figure 1**) include: oligosaccharides, phenolic compounds, carotenoids, alkaloids, terpenoids, saponins, phytosterols and others (Huang et al., 2016).

### 2.1 Phenolic compounds

One of the most notable and studied class of phytochemicals are phenolic compounds (PC), which from a chemical perspective are characterised by one or more hydroxyl groups (-OH) attached to an aromatic hydrocarbon group. Phenol (C_6_H_6_O) stands as the fundamental chemical structure on which this group (ranging from simple phenolics to complex and highly polymerized compounds) is based (Vermerris and Nicholson, 2006). Through LC-MS and GC-MS identification, PC were by far the most frequently described phytochemicals in legume varieties with phenolic acids, flavonoids and condensed tannins the primary sub-classes found in seeds (Tor-Roca et al., 2020).

Phenolic acids are relatively small molecules when compared to other PC such as flavonoids or tannins, and they are characterised by a phenol with a carboxylic acid functional group (Vermerris and Nicholson, 2006). They include hydroxybenzoic acid derivatives, gallic, vanillic and syringic acids and hydroxycinnamic acid derivatives such as caffeic, ferulic, p-coumaric and sinapic acids (Singh et al., 2017). Flavonoids, in turn, stand out as one of the most prevalent group of PC which is particularly abundant in various types of legumes (Tor-Roca et al., 2020). With two benzene rings linked together by a group of three carbons they are divided into anthocyanin- (coloured), and anthoxanthin- (colourless) compounds. Anthoxanthins are divided further into flavones, flavans, flavonols, and isoflavones, the latter almost exclusively found in leguminous plants (Mutha et al., 2021). Daidzein and genistein, major isoflavones present in soybean (*Glycine max*), have been linked to reduced risk of certain types of cancer, namely prostate (Adjakly et al., 2013), and breast cancer (Sohel et al., 2022).

Tannins are a class of compounds that have a wide structural diversity and share the ability to conjugate with polysaccharides and proteins. They are usually divided into two groups: hydrolysable- or condensed-tannins, with the former being able to undergo hydrolysis, and produce carbohydrates and phenolic acids. In contrast, condensed tannins are more stable and obtained through the condensation of flavan-3-ols (Hill, 2003). Like almost all PC, tannins have shown antioxidant properties and bioactive potential. Nevertheless, the complexes formed between tannin-protein can reduce protein digestibility and inactivate digestive enzymes, thus the perception of these compounds as (solely) ‘anti-nutritional’ factors (Samtiya et al., 2020). Regardless, it is important to assess their health benefits alongside possible undesired effects (Geraldo et al., 2022).

Lignans are part of a group of diphenolic compounds and have been extensively studied for their health benefits due to their steroid-analogous chemical structure (Senizza et al., 2020). They share some chemical and structural characteristics with lignin which is a phenolic polymer found in the cell walls of legumes and other plants, and it is the third most abundant biopolymer on Earth (Pérez et al., 2002; Vermerris and Nicholson, 2006). As a complex polymer possessing a cross-linked structure, lignin is relatively resistant to mineralisation or decomposition, and can be found in high concentrations in woody tissues, contributing to both plant- and soil-health (Frei, 2013).

The biological activities of PC are frequently centred on extractable phenolic compounds, meaning compounds that can be easily extracted from the matrix using water or organic solvents, or a mixture of these. Yet, it is now recognized that this approach overlooks the non-extractable PCs or bound phenolics - a considerable proportion of dietary polyphenolics, which are not extracted using traditional methods (Silva et al., 2018). Several legume crop species have been shown to possess high amounts of both PC and dietary fibre (polysaccharides) in the food and in-field residue components, which in turn leads to bound phenolics in their matrices. Legume PCs interact with each other and other compounds, such as carbohydrates, proteins, or lipids, through reversible noncovalent and mostly irreversible covalent interactions, impairing the bioavailability of these bound phenolics (Rocchetti et al., 2022).

### 2.2 Alkaloids

Alkaloids are a diverse group of chemicals that possess alkali-like properties and consist of at least one N atom within a heterocyclic ring structure. While not all legumes contain alkaloids, some species are known to produce these compounds, with significant contribution to plant growth and defence. For instance, faba beans and peas have residual alkaloids concentrations but, quinolizidine alkaloids are common and well-documented among commercially important lupin genotypes. They have garnered significant attention from farmers and breeders due to their potent bitter taste and potential toxicity at high doses (Pereira et al., 2022), which is why it is recommend they remain below the industry threshold of 0.02% (Boschin et al., 2008). Nevertheless, recent studies show that lupanine, one of the prevalent alkaloids of the genus *Lupinus* in both their foliage and their seed (Hill, 2003), has been shown to have anti-inflammatory and anti-cancer properties (Guerra-Ávila et al., 2023). Sparteine, another lupin alkaloid, has been associated with antiarrhythmic and anticonvulsant properties (Frick et al., 2017), showcasing the dual nature of alkaloids in legumes as both anti-nutritional factors and potential pharmaceutical agents.

### 2.3 Saponins

Saponins (a group of terpenoids) in legumes are present in the form of triterpenoid glycosides or steroids that have a variety of biological activities (Mustafa et al., 2022). Due to their surfactant character, they have the ability to produce foam, and are often responsible for the soapy taste and bitter flavour of some legume species (Shi et al., 2004). Some of the most studied saponins in legumes include soyasaponins, which are found in soybeans and other members of the legume family, such as lentil and pea (Tor-Roca et al., 2020). These compounds have been shown to have a range of biological activities, including cholesterol-lowering effects, anti-cancer properties, and anti-inflammatory effects (Guang et al., 2014). However, in addition to their bitter taste, saponins can be toxic when present in high concentrations and may exert adverse effects on nutrient absorption. This is primarily due to their capacity to inhibit metabolic and digestive enzymes, as well as their tendency to bind to essential nutrients like zinc (Samtiya et al., 2020), emphasising the trade-off that we have with most of these phytochemicals between positive and disadvantageous traits.

### 2.4 Phytosterols

Legumes are also a high dietary source of phytosterols (steroid compounds derived from plants), and these comprise the bulk of unsaponifiable substances found in plant lipids (Salehi et al., 2020). Structurally similar to cholesterol, they are thought to have cholesterol-lowering effects (Gupta et al., 2011), hence they are regarded as important dietary elements for upholding good cardiovascular health. Lentil hulls have been found to contain elevated concentrations of phytosterols, namely β-Sitosterol (Mustafa et al., 2022), which has potential as a notable drug of the future due to their action as a neuroprotective and chemopreventive agent (*i.e.*, preventing, reversing of blocking invasive cancers) (Babu and Jayaraman, 2020).

### 2.5 Phytic acid

Phytic acid, also known as phytate, myo-inositol hexakisposphate or IP6, is present in several legumes but its concentration can vary greatly among pulses, with considerable discrepancies between high-phytic acid species as common bean or pea and low-phytic acid species, like faba bean and lentils (Sinkovič et al., 2023). Phytic acid can also complex both micro- and macro-elements, potentially leading to a reduction in the bioavailability of minerals, protein function, and ion cofactors to ensure proper enzymatic function (Shi et al., 2018). When consumed within the recommended daily doses, nonetheless, phytates have important health benefits, either chelating toxic metals or through anticarcinogenic properties and antioxidant activity (Geraldo et al., 2022). The majority of the phosphorus contained within seeds exists in the form of phytic acid (Gupta et al., 2015).

### 2.6 Raffinose family of oligosaccharides

Oligosaccharides from the raffinose family, also known as α-galactosides, have bonds between galactose residues ranging from one to six. In humans, the enzyme needed to break down these compounds is not found in the small intestine. As a result, consuming grains like fava beans and peas (Carbas et al., 2021), which contain these oligosaccharides, often leads to excessive quantities of intestinal gas, and stomach discomfort (Pereira et al., 2022). Despite these unwanted side effects, legume-derived raffinose family oligosaccharides are increasingly being included in pro- and pre-biotics. They also show potential in combating obesity and diabetes and preventing non-alcoholic fatty liver disease (Elango et al., 2022).

## 3 Soil- and gut microbes and functional diversity concept

Soil and gut microbiomes are complex and diverse ecosystems that play vital roles in various biogeochemical processes, while contributing significantly to both animal and human health. Soils represent the largest repository of organic matter and biological diversity on Earth, home to a vast array of microbial communities characterised by high functional diversity that play crucial roles in soil fertility, nutrient cycling, and plant health (Mishra et al., 2023). However, soil is a non-renewable natural bio-resource, that had to carry the relentless burden of intensified agricultural production to meet the rapid increase of the human population and rising consumption levels. With food demands estimated to increase up to 70% by 2050 (Giller et al., 2021), a shift from conventional or intensive high agrochemical input agriculture (which severely contributes to climate change through GHG production, eutrophication through N- and phosphorous-losses, including via soil loss, and soil function) to regenerative agriculture is of increased interest (Newton et al., 2020). Common regenerative agriculture practices include reduced synthetic (nitrogen) fertiliser use, adoption of intercropping systems (Brooker et al., 2015) and reduced tillage, well-known practices that are increasingly implemented. Nevertheless, soil microbiome manipulation in regenerative agriculture should be given due consideration as an underlying tool for providing various essential benefits to soil (Hermans et al., 2023).

On the other hand, gut microbiome refers to the diverse and active microbial communities inhabiting the gastrointestinal tract of humans and animals. Although the ratio is likely to vary among individuals, the gut microbiome constitutes a significant niche of overall human microbiota (Santhiravel et al., 2022), typically maintaining a 1:1 ratio with the number of human cells (Walker and Hoyles, 2023). Known for its high functional diversity, which is attributed to the multiplicity of microorganisms inhabiting, gut microbiome is key to human health, with a critical role in digestion, modulation of the immune system, barrier function against pathogens and metabolism (Sudheer et al., 2022).

The concept of ‘functional diversity’ within a microbiome encompasses the vast range of purposes that a community of microorganisms can perform, beyond simply the types of microbes that are present, since both soil and gut microbes’ function is crucial for maintaining the sustainability of natural ecosystems and the health of individuals, respectively (Blum et al., 2019), with certain aspects of this functional diversity of soil and gut microbes being impacted by legume phytochemicals in various ecological and physiological processes.

It should also be noted that the influence these distinct phytochemicals have on microbial ecology could be substantially different; and, even within the same class of compounds, mechanisms of action vary. Taking flavonoids as an example, an influential group of phytochemicals (Mutha et al., 2021), their influence on the gut microbiome appears to involve distinct mechanisms that differ from their effects on the soil microbiome. While in the soil, flavonoids promote rhizobia movement and enhance nodule organogenesis (Wang et al., 2022), their actions within the gut exhibit diverse pathways, fostering symbiotic relationships, modulating the growth of beneficial bacteria in the gut and potentially contributing to the overall health of the host organism (Pei et al., 2020). Such nuanced disparity underscores the multifunctionality of flavonoids, showcasing their differential impact on distinct microbial ecosystems. In the next section, we discuss the specific impacts of different fractions of phenolic compounds and other legume phytochemicals on soil and gut microbial communities.

### 3.1 Legume phytochemicals impact on the functional diversity of soil microbes

With agroecological outcomes ranging from local (impacts on soil health and fertility) to global scales (emission of greenhouse gases) (Berihu et al., 2023), the soil microbiome and its interaction with plants and their phytochemicals is of upmost importance, due to their potential to support sustainable and regenerative agriculture, and downstream food- and feed-systems.

**Figure 2:**
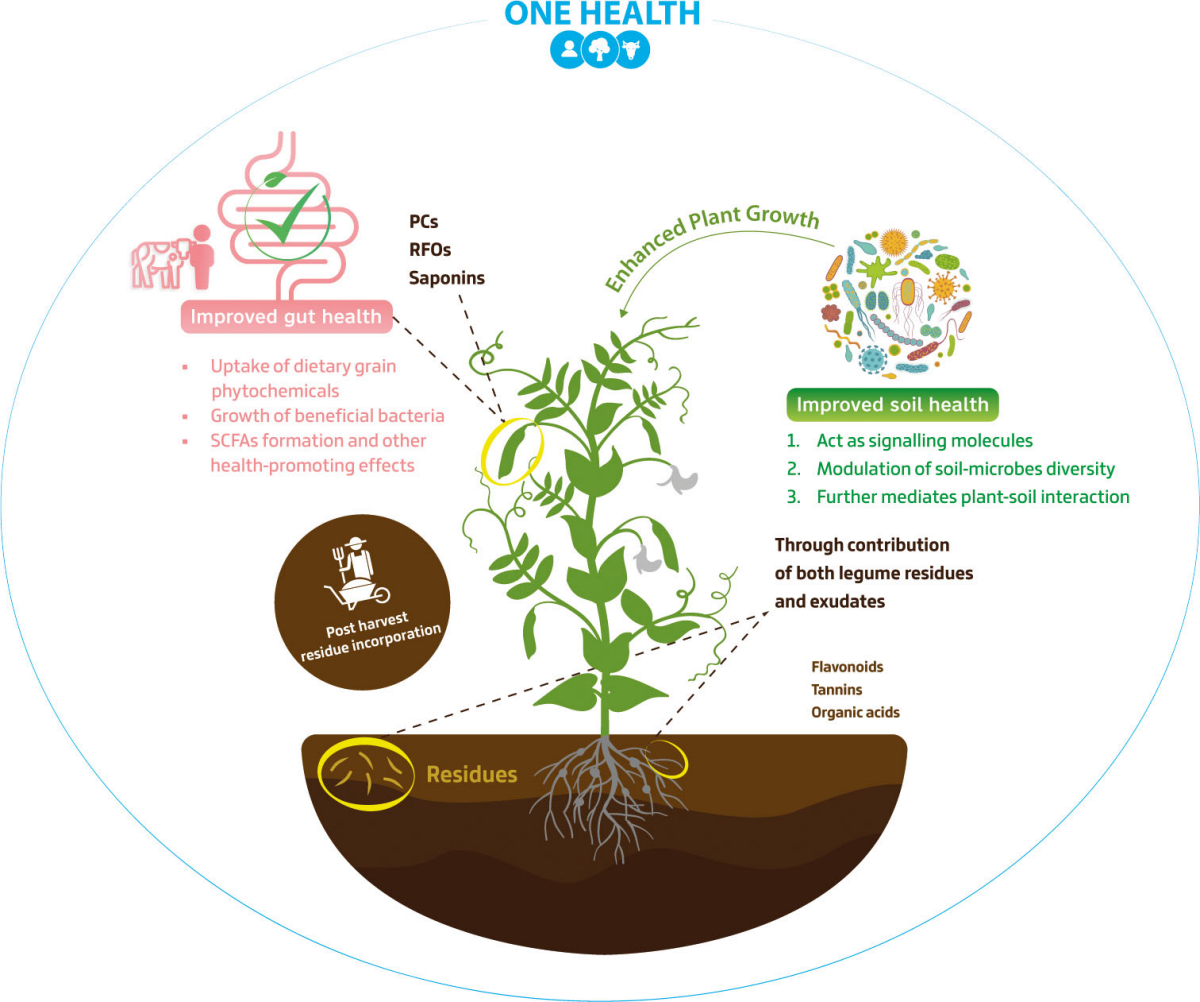
Soil and gut microbiomes as impacted by legume phytochemicals- One Health Approach. This figure explores the interplay between soil and gut microbiomes influenced by legume phytochemicals. The diverse impact of phytochemicals, e.g., phenolic compounds (PCs) and raffinose family oligosaccharides (RFOs), is depicted, encompassing factors such as short-chain fatty acids (SCFAs) production and exudate molecules influence on soil microbial diversity. Notably, ‘Post-harvest residue incorporation’ section emphasizes the significance of integrating legume residues into the soil after harvest, fostering microbial activity and nutrient recycling.

The symbiotic relationship between legumes and soil bacteria is a well-established and vital component of sustainable agroecosystems. To study the rhizosphere, the soil zone influenced by plant roots, researchers use techniques such as DNA-based assays or transcriptomics to identify changes in microbial communities and gene expression. In addition, rhizobia are one of the most studied soil microbe types considering their role in initiating nodule formation in the roots (or stems) of legumes, where the aforementioned BNF occurs. This process involves a series of biochemically directed steps, which includes colonization and nodule organogenesis, where various phytochemicals come into play, with flavonoids being one example. When released by legume roots as component of their exudate, flavonoids can act as chemo-attractants, facilitating the movement of compatible rhizobia (**Figure 2**) towards the roots and enhancing the colonization and infection process, root nodule formation and so the efficiency of BNF (Cooper, 2004; Abdel-Lateif et al., 2012). Conversely, legume flavonoids may exhibit antimicrobial activities that influence the overall recruitment and functioning of the root microbiome. This negative effect on specific taxa recruitment, for example on parasitic nematodes, fungus, and pathogenic bacterial species (e.g. *Pseudomonas mars-prunorum* and *P. phaseolicola*) (Shah and Smith, 2020) highlights the complex interplay of these different mechanisms of action, underscoring the intricate relationship between legume phytochemicals and microbial populations in the soil. Additionally, flavonoids can even act as regulators of nodulation (nod genes) in rhizobia, which are essential for producing species-specific Nod factors. These factors, in turn, trigger specific changes in plant tissue, such as root cortical cell division and root hair curling, which are critical modifications for nodule organogenesis (Hassan and Mathesius, 2012). The ability to modulate certain microbial communities through flavonoid metabolism is an area of increasing interest. This research has potential applications, including extending nodulation to plants beyond legumes (Abdel-Lateif et al., 2012) or engineering plant phytochemicals to influence beneficial microbiomes (Wang et al., 2022).

Phenolic acids are also commonly presented by legume roots as exudate, and may accumulate in soil, to be used as a source of carbon by several rhizobia species, influencing legume-microbe symbiosis (Seneviratne and Jayasinghearachchi, 2003). In some instances, specific taxa as Rhizobia and Pseudomonas, have been identified as capable of breaking down flavonoids compounds into simpler structures, namely phenolic acids (Wang et al., 2022). For example, protocatechuic acid was found to be responsible for the indirect allelopathic effects of catechin, a flavonoid (Wang et al., 2014). Research suggests that the accumulation of phenolic acids led to an increased relative abundance of Bacteroidetes and Firmicutes, which utilize these compounds as growth substrates (Bai et al., 2019), and have been correlated with detrimental impacts on soil micro-ecological environment. An increase in Pseudomonas population, on the other hand, was shown to promote plant growth while degrading phenolic acids present in the rhizosphere (Wang Y. et al., 2021). These findings collectively highlight that the accumulation of phenolic acids alters microbial community structure, even as the specific mechanism of influence and their consequential effects on soil ecology and plant growth remains unclear.

Although most research has focused on the regulation by flavonoid signalling rather than by phenolic acids, there is growing evince for phenolics as determinants of nodulation. A recent study based on ultra-high-performance liquid chromatography-tandem-quadrupole mass spectrometry has reported an increased plant growth and number of nodules in *L. japonicus*, due to the action of three phenolic acids (p-coumaric, caffeic, and ferulic) enhancing *nod* genes expression, thus being considered a novel type of nod inducers (Shimamura et al., 2022). The wide range of phenolic compound classes reflects their multifaceted impact on the soil microbial community, which is important to prevent or mitigate dysbiosis in soil microbiomes that would then affect plant yield and overall nutritional quality (Giovannetti et al., 2023). Given the close link between plant nutrition and human health (Hoffmann et al., 2022), this foreshadows the importance of legume phytochemicals with microbe-modulating properties to support the One Health approach (**Figure 2**).

Besides phenolics, organic acids are key determinants on soil microbiome structure and function. It was reported in a metagenomic approach that organic acid exudation by legume roots, when compared to wheat, explained 15% of the observed variance in rhizobacterial abundance in a microcosm (Schaedel et al., 2021). Small molecules like malic or oxalic acid, impact not only microbial activity, and are also capable of improving soil fertility too such as via mobilizing less-labile forms of P (Yu et al., 2021), promoting nutrient cycling, soil structure, and overall ecosystem resilience. Legume symbiosis with other soil microbes, such as arbuscular mycorrhizal fungi (AMF), is also highly influenced by specific molecules in root exudates. AMF are natural root symbionts, forming symbiotic relationships with a vast number of plants and helping to increase inorganic phosphorus acquisition (Wang et al., 2022). Such enhancement of root performance is in-turn helpful to nodules as they have high demand for energy (ATP-consuming), and so inorganic phosphate too. Some types of flavonoids are capable to stimulate AMF colonization, such is the case of quercetin (Tian et al., 2021) and isoflavones (daidzein and genistein) (Sugiyama, 2019), both present in root exudates, leading to an overall increase of plant biomass. Phenolic acids (Mandal et al., 2010) and strigolactones (group of sesquiterpenes) can also stimulate germination of spores and hyphal growth (Steinkellner et al., 2007), being another example of chemical-induced shifts in plant-microbial interactions leading to functional changes in the ecosystems.

Recent discoveries regarding soyasaponins, a type of saponin found in the root exudates of legume species, have highlighted their functional potential too. These compounds can disperse over greater distances in the rhizosphere, compared to isoflavones. They can influence bacterial communities by increasing the populations of bacteria that promote plant growth, such as *Novosphingobium* which induces the breakdown of organic matter in soil leading to the production of nutrients that plants can uptake via their roots (Fujimatsu et al., 2020). Phytochemicals, acting as antioxidants and metal chelators, also have the capacity to regulate microbial activity and soil properties, including the formation of soil organic matter and nutrient cycles (Vukicevich et al., 2016). For example, certain phytochemical-rich legume species, impact the availability of soil minerals by chelating and oxidizing/reducing metals. Isoflavonoids found in alfalfa can dissolve ferric phosphate, making both phosphate and iron accessible to the plant (Hassan and Mathesius, 2012). Tannins, on the other hand, have been shown to counteract the toxic effects of some metals through chelation, thereby positively influencing soil function and plant health (Halvorson et al., 2012). Conversely, alkaloids especially present in lupin crops can potentially have a detrimental environmental impact on plant-associated microbiota due to their mobility in soil environments. They have been recently identified as aquatic micropollutants (Günthardt et al., 2018; Hama et al., 2022).

Overall, the impact of legume phytochemicals on the soil microbiome structure and function can be complex and depend on the specific compounds produced by the legume, as well as the microbial populations present in the soil. Further research should prioritise the study of legume phytochemicals to better comprehend their influence on soil microbial relationships. Consequently, this understanding can enable the selection of select specific phytochemical-rich legumes that selectively promote the growth of beneficial microbial populations and/or soil functions of agroecosystems.

#### 3.1.1 Legume phytochemicals impact as part of a residue management strategy

Currently, there is a growing emphasis on production systems that prioritise the sustainable management of natural resources to meet the needs of both present and future generations. This objective aligns the increased cultivation of cover crops, that is, species mixtures which usually include a high-density of legumes, sown before or after cash-crops, to cover and protect the soil between main harvest seasons, and serve as a plant-based manure (Kocira et al., 2020). By utilising cover crops effectively, European farmers can contribute to achieving the EU mission’s goals and fostering more sustainable and resilient agricultural systems (Fendrich et al., 2023). When applied efficiently legumes, such as clover, vetch, and alfalfa, are popular choices for cover cropping (Scavo et al., 2022), considering their lower C:N ratio and high levels of biomass produced. Nevertheless, legumes cover crops are more than merely good sources of bio-accumulated N for the following crop, as they present several phytochemicals that can impact the decomposition or mineralisation rates of their residues - and which overall improve serve as a ‘nature-based solution’ (Cohen-Shacham et al., 2016), to enhance soil health, crop-protection and increase productivity (**Figure 2**). For instance, tannins which are commonly found in forage legumes, are a group of polyphenolic compounds that can inhibit microbial decomposition, leading to a longer-lasting residue cover on the soil surface. This can offer protection against erosion, reduce weed growth, and enhance moisture retention (Halvorson et al., 2012; Erktan et al., 2017). Although not exclusive to the *Fabaceae* family, legumes show a greater lignin content than grasses, which also contributes to the persistence of residues in the soil and the aforementioned beneficial traits (Döring et al., 2013). Recently, cover crops residues (often called green manure) were also found to possess various antimicrobial secondary metabolites such as quercetin (a flavonoid) that help reduce the population of *Escherichia coli* in contaminated agricultural soils (Zhao et al., 2023) and although not applied on the soil saponins extract from soybean residues have shown a eco-friendly potential for washing pesticide residues off fresh-cut vegetables and fruits industry (Hsu et al., 2022).

Soil is a very complex system and the interplay between biotic and abiotic stresses has an enormous influence on how the regulation and signalling of these phytochemicals occurs. For instance, it has been established, that flavonoids present in root exudates and cover crops residues are critically involved in several different pathways that mediate plant-soil interaction. However, in soils where an excessive amount of organic matter accumulates, this can result in a dampening of flavonoid signalling, which in turn, can reduce important ecological processes like nodulation and overall plant growth (Del Valle et al., 2020).

In conclusion, the intricate dynamics within the complex soil system highlight the continued significance of legume phytochemicals study on microbiome modulation (**Table 1**). Moreover, these shifts on soil microbial diversity may result in a transmission of soil-endemic microbes, leading to a more diverse gut microbial structure and distinct bacterial composition in people closely associated with gardening activities, a primary source of soil contact, in contrast with a non-gardener group (Brown et al., 2022). As gut and the soil microbiome share similar bacteria phyla (*Bacillota, Bacteroidota, Pseudomonadota*, and *Actinomycetota*) and they can act interlinked on human health, it is important to avoid poor soil maintenance/practices, as healthy soils often lead to healthy guts (Blum et al., 2019).

**Table 1:** Legume phytochemicals effect on soil microbiome structure and function.

Phytochemical	Common source	Microbiome structure	Microbiome function	Reference
**Flavonoids**	Ubiquitous in legumes (exudates)	Promote rhizobia movement toward the roots	Enhanced nodule organogenesis	(Cooper, 2004; Abdel-Lateif et al., 2012)
		↑ Arbuscular mycorrhizal fungi	Stimulates symbiotic relationships and overall increase of plant biomass	(Sugiyama, 2019)
		↓ population of *Escherichia coli*	Bio mitigation strategy to improve on-farm produce safety	(Zhao et al., 2023)
**Tannins**	Forage legumes	Inhibit microbial decomposition	Longer-lasting cover on the soil surface reducing erosion and weed growth	(Halvorson et al., 2012; Erktan et al., 2017)
**Phenolic acids**	Ubiquitous in legumes (exudates)	Source of carbon by selective rhizobia species	↑ Legume-microbe symbiosis	(Seneviratne and Jayasinghearachchi, 2003)
**Saponins**	Widely found in legumes	↑ *Novosphingobium*	Production of nutrients that can be absorbed by plant roots	(Fujimatsu et al., 2020)
**Alkaloids**	Lupin species	Toxic properties to soil microbial communities.	Leaching from agricultural fields lupin alkaloids are associated as aquatic micropollutants	(Hama et al., 2022)
**Lignin**	Ubiquitous in legumes	Selection of saprotrophic microbiota	Persistence of residues in the soil increasing N soil accumulation	(Döring et al., 2013; Bonanomi et al., 2019)
**Organic acids**	Ubiquitous in legumes (exudates)	Associated rhizobacterial diversity	Improving soil fertility	(Schaedel et al., 2021; Yu et al., 2021)

Direction of arrows denotes to an increase (↑) or decrease (↓) relative to the discussed element.

### 3.2 Legume phytochemicals impact on the functional diversity of gut microbes

When viewed through the lens of the One Health approach (**Figure 2**), it is clear how legumes are key drivers of human, animal, and environmental health, emphasising that the health of one component directly impacts the others (Mackenzie and Jeggo, 2019). Nevertheless, for such a complex system to thrive, it has to be resilient enough to adapt long-term human interference (Rendon et al., 2020). Considering that, ‘functional redundancy’ has often been regarded as one of the promoters of ecological resilience, either through communities with redundant species (those that perform similar functions) (Biggs et al., 2020) or even metabolic networks that have evolved to have redundant genes for key metabolic pathways (Sambamoorthy and Raman, 2018). In this context, we propose that legume phytochemicals also exhibit a degree of redundancy, as it was already discussed that various classes of compounds fulfil analogous roles. This redundancy is crucial to the capacity of legumes and their phytochemicals to serve as a pivotal bridge to enable better choices to both environmental and human dietary concerns.

Acting within the intestine, legume phytochemicals modulate gut microbiota structure and function through several cellular signalling pathways, with absorption and metabolism by the gut microbiome being a complex topic and an area of active research (Sudheer et al., 2022). Taking PC or RFOs as examples, some are absorbed in the small intestine and enter the bloodstream, but many others (due to their complex chemical structure) pass to the colon where gut bacteria metabolize them. There, a variety of enzymes can break down the parent compounds into smaller molecules and intermediates that are more easily absorbed into the blood stream (**Figure 2**). These metabolites can have different biological activities compared to the original compounds, and some may have even stronger health-promoting effects (Santhiravel et al., 2022). *In vitro* models of the human gastrointestinal tract are key tools to simulate normal conditions, and study how these phytochemicals interact with gut microbes (Qi et al., 2023). Additionally, the utility of such models can be extended when allied with techniques such as high-throughput sequencing to discern the genetic basis of underpinning mechanisms of different phytochemicals. For instance, gut antioxidant function can be associated with both PC and carotenoids (Singh et al., 2017), while lignans and/or flavonoids can interact with hormonal receptors due to their hormone-like and anti-hormone effects (Liu et al., 2022). It is also important to mention that some phytochemicals, like phenolics (Manco et al., 2023), are more present in the hulls (i.e. seed coats of legume grains) compared to kernels (i.e. seeds without coats), which are isolated after dehulling (or decortication). The hulls are a coproduct which is often processed for use as a significant component of ruminant and pet-food diets (Halmemies-Beauchet-Filleau et al., 2018), but could contribute to a better health, considering the biological properties of their rich phytochemical profile.

#### 3.2.1 Prebiotic effect

More than two decades ago, a certain group of non-digestible compounds (although selectively hydrolysed by gut microbes) were acknowledged for their capacity to enhance the microbiota and health of the host organism and became known as ‘prebiotics’ (Davani-Davari et al., 2019; Voss et al., 2022). Most prebiotics are also phytochemicals and the first recognised as such were oligosaccharides, as RFOs (Gibson et al., 2017). Raffinose family oligosaccharides, as well as sucrose, are the primary soluble carbohydrates present in legumes (Carbas et al., 2021; Elango et al., 2022). Their influence can be observed through various distinct effects and thanks to metabolomic analysis in specific, researchers have been able to identify and quantify such small molecules produced during the interaction of prebiotics with gut microbes (Rodríguez-Daza et al., 2021). Ciceritol, a common oligosaccharide of chickpea, was reported to enhance the growth of beneficial bacteria, such as *Lactobacillus, Enterococcus* and *Bifidobacterium* spp., while boosting the production of short chain fatty acids (SCFAs) (Zhang et al., 2017), key components of a healthy gut (Gomes et al., 2022). Similarly, lupin kernel RFOs such as stachyose and verbascose, have the potential to increase the *Bifidobacterium* population in the colon (Martínez-Villaluenga et al., 2005; Pereira et al., 2022). Note too, that there is often overlap between different phytochemical categories, and many compounds may have multiple and similar properties and functions in the body. Legumes intra-specific phytochemical diversity, home cooking, and industrial processing treatments also affect significantly *in vitro* digestibility of food- and feed-stuffs. So, it is important to consider these parameters when assessing gut microbiota structural shifts, and SCFAs production (Chen et al., 2020). Recently added to the prebiotic concept, PC also have the capacity to stimulate selective beneficial bacteria (Rodríguez-Daza et al., 2021), e.g. *Eubacterium* spp., *Lactobacillus* spp., *Akkermansia* spp., *Bifidobacterium* spp., *Clostridium* spp. and *Bacteroidetes* spp (Queiroz-Monici et al., 2005; Moreno-Indias et al., 2016; Lordan et al., 2020; Calderón-Pérez et al., 2021; Dong et al., 2022; Rocchetti et al., 2022; Rodríguez-Daza and de Vos, 2022).

#### 3.2.2 Effect of dietary phytochemicals on disease

Good gut health relies on the optimal structure and functioning of its microbial community. The diverse range of microorganisms residing in the gut is acknowledged as a key factor in determining the level of risk from health conditions such as cancer (Hanahan, 2022), obesity (Liu et al., 2021), diabetes (Zhou et al., 2022), and inflammatory bowel diseases (Wang L. et al., 2021). As mentioned in the earlier section, phytochemicals have shown significant effectiveness in altering the composition of the intestinal microbiota, which in-turn affects an individual’s susceptibility to diseases and their response to them through various pathways. After changes in gut microbiome diversity (in terms of the balance and richness of different species), there’s also an impact on its function.

Compounds such as flavonoids, for instance, have been shown to increase the population density of *Akkermansia muciniphila* in the gut. This increase has been associated with protective effects against obesity development, and the improvement of the gut-blood barrier’s integrity (Juárez-Fernández et al., 2021). Similarly, the gut microbiota also plays a role in the production of equol, a metabolite of legume isoflavones, which is generated by bacteria such as *Adlercreutzia equolifaciens* and *Bifidobacterium bifidum*. Individuals who produce equol have been linked to better lipid metabolism and a lower prevalence of dyslipidaemias (high cholesterol) (Zheng et al., 2019), as well as protection against autoimmune encephalomyelitis (inflammation of the brain and spinal cord) (Jensen et al., 2021) and cardiovascular diseases (Pilšáková et al., 2010). Another isoflavone, genistein, is metabolised by gut bacteria and has been found to improve insulin signalling and reduce fat accumulation in the liver, a known risk factor for obesity and type 2 diabetes (Arunkumar et al., 2013). On the other hand, it has been reported that soy isoflavone-rich extracts do not significantly reduce lipid accumulation in cell cultures, but do seem to have anti-inflammatory potential by decreasing TNF-α (tumour necrosis factor alpha) and increasing IL-10 (an anti-inflammatory cytokine) (Angelotti et al., 2020).

Phytochemicals from various legumes, when processed by intestinal microbiota, can produce similar effects while generating different intermediate compounds. This is evident in the case of saponins. In lentil extracts, saponins are transformed into sapogenins, which have the ability to influence the growth of specific intestinal bacteria (Navarro del Hierro et al., 2020). On the other hand, soyasaponins, which are commonly found in soybean, have been observed to reduce inflammation caused by allergies by positively impacting the composition of the intestinal flora (Nagano et al., 2017). Additionally, other bioactive compounds, like β-sitosterol, a phytosterol commonly present in legume ingredients found in Mediterranean diets (Jiménez-Escrig et al., 2006), have demonstrated the capability to inhibit certain bacterial genera that possess the genes responsible for a major pathway leading to atherosclerosis (the build-up of lipids in arteries) (Wu et al., 2022). Plant sterols have also been associated with an increase in the abundance of *Eubacterium hallii* (Cuevas-Tena et al., 2018), an anaerobic bacterium currently classified as *Anaerobutyricum hallii*, known for producing butyrate, which has positive implications for insulin sensitivity treatments in obese and diabetic mice (Udayappan et al., 2016).

While lignans are typically classified as polyphenols, they have a chemical structure resembling steroids, akin to phytosterols. Consequently, they are referred to as natural phytoestrogens. The inherent bioactive potential of the parent compound is limited, but when certain bacteria capable of hydrolysing lignans, such as *Lactobacillus* spp. and *Bifidobacterium* spp., act on them, they are transformed into enterodiol and enterolactone. Enterolignans share structural similarities with estrogen steroid hormones and function as natural ligands for estrogen receptors. They have the potential to reduce LDL (low density lipoprotein) cholesterol levels and lower the risk of cardiovascular diseases (Creus-Cuadros et al., 2017; Senizza et al., 2020).

Phytochemicals may also have potential anticancer effects. Bacterial transformation of lignan was reported to alleviate tumour burden in a rat model of breast cancer (Mabrok et al., 2012) and inhibited the proliferation of prostate tumour cells (Sepporta et al., 2013), with underlying mechanisms requiring further investigation. Also, highlighting the protective impact of a diet rich in lignans, urinary enterolactone levels were inversely associated with ‘all-cause mortality’ (death due to any disease, complication, or hazardous exposure), in a prospective study conducted on over 6000 adults (Liu et al., 2020). However, impact on the functional diversity of gut microbiota lacks long-term human studies and follow-ups on short-term dietary intervention.

Overall, gut microbiome shifts related with the metabolism of phytochemicals have important implications for human health, since communication between different organs and the gut is paramount. Moreover legumes are a vast family of plants with an even wider range of phytochemicals and although there is some research in this area, a full evaluation of the genetic and phytochemical diversity offered by legume crops remains to be fully elaborated, with potentially phytochemical-rich underutilised crops or legume processing by-products also requiring more detailed investigation (Ebert, 2014). In fact, when we explore the extensive effects of legume dietary compounds on human health, it naturally prompts us to consider broader implications, including how these legume phytochemicals might have similar effects on the microbiomes of different animal species (Sherasia et al., 2018).

#### 3.2.3 Animal gut microbiome

Livestock accounts for approximately 40% of the total value of agricultural production worldwide (Kulkarni et al., 2018) and despite new influx of public policies for the inclusion of legume grains as protein sources, the European Union still heavily relies on high protein grain imports to meet their livestock (and aquaculture) protein requirements (Jouan et al., 2020). For the last few decades, legume incorporation in animal feed forages was often discouraged due to their impact on animal gastrointestinal function via factors like tannins. Tannins, specially condensed tannins, are common molecules found in forage legumes and as previously discussed, play a significant role on astringency and bitter taste. Although, oral sensitivity in ruminant species is not well documented, this process can lead to a decrease in preference and eventually a reduction of forage consumption by some livestock species (Lamy et al., 2011). Moreover, the two most cultivated forage legumes, which happen to be low in tannins, white clover and lucerne, have been related with bloat^1^, a common digestive disorder in ruminants that causes damage to gut function (Caradus et al., 2022). On the positive side, legume species presenting high levels of tannin can inhibit the activity of rumen microorganisms and biofilm development, and slowing initial rates of digestion which is a distinguishing bloat-causing feature (Wang et al., 2012). Furthermore, tannins have the ability to reduce the negative impacts of gut parasites, such as for example gastrointestinal nematodes, by inhibiting the activity of key parasite enzymes (Mueller-Harvey et al., 2019). Legume tannins can also reduce methane emissions in agricultural systems directly or indirectly, by either inhibiting gut methanogens (methane producers) or/and protozoal (generates intermediates used by methanogens) populations (Ku-Vera et al., 2020; Caradus et al., 2022). Consequently, the ‘anti-nutritional’ view of phytochemicals (as is the case for tannins) is shifting to the point where they are now often considered beneficial for ecological and physiological wellbeing - reducing GHGs emissions and the impacts of climate change, and protecting ruminant health, when consumed in adequate amounts (Singh and Bhat, 2001).

Legume saponins have also been described as toxic to gut protozoa (Ramos-Morales et al., 2017) therefore lowering methane outputs. Additionally, flavonoids, well-known by now for their anti-inflammatory and antimicrobial properties, are believed to improve overall productivity and growth of ruminants (Ku-Vera et al., 2020). Most beneficial in periods of stress, flavonoids can also select against Gram-positive microorganisms leading to an increase in the production of propionate (compared to acetate), increasing the efficiency of ruminant digestion and so performance (Olagaray and Bradford, 2019).

These examples illustrate how legume phytochemicals are not only significant to human health but can also highly influence animal gut microbiome-ecology and -function (**Table 2**). However, more studies are needed to better understand the impact of other secondary metabolites, such as terpenes and alkaloids (Tedeschi et al., 2021).

**Table 2:** Legume phytochemicals effect on gut microbiome structure and function.

Phytochemical	Common source	Microbiome structure	Microbiome function	Reference
**Flavonoids**	Ubiquitous in legumes	↑ *Akkermansia muciniphila*	Protective effect in obesity development and enhancement of gut barrier integrity	(Juárez-Fernández et al., 2021; Rodríguez-Daza and de Vos, 2022)
		Select against Gram-positive microorganisms	Increase in the production of propionate in ruminates	(Olagaray and Bradford, 2019)
**Tannins**	Forage legumes	Inhibit methanogens rumen microorganisms	Prevents bloat, reduces methane emissions	(Singh and Bhat, 2001; Kulkarni et al., 2018)
**Phenolic acids**	Ubiquitous in legumes	↑ Bacteroides and SCFAs production	Mitigate intestinal inflammation	(Hwang et al., 2022)
**Isoflavones**	Soybean	Selectively metabolized by *Adlercreutzia equolifaciens* and *Bifidobacterium bifidum*	Anti-inflammatory potential and improved insulin signalling (reduced risk factors of obesity and type 2 diabetes)	(Arunkumar et al., 2013; Zheng et al., 2019; Mutha et al., 2021)
**Saponins**	Forage legumes	Ruminal protozoa elimination	Improved N-use efficiency and ruminant animal performance	(Olagaray and Bradford, 2019; Kholif, 2023)
**Ciceritol**	Chickpea	↑ *Lactobacillus* and *Bifidobacterium* spp.	Increased production of short chain fatty acids (SCFAs)	(Zhang et al., 2017)
**Lignans**	Ubiquitous in legumes	Reduce to enterolignans by lignan-hydrolysing taxa	Lower LDL cholesterol through the production of natural ligands of estrogen receptors	(Creus-Cuadros et al., 2017; Liu et al., 2020; Senizza et al., 2020)
**Phytosterol**	Pulses	↑ *Anaerobutyricum hallii* (former *Eubacterium hallii)*	Increased butyrate-production linked to insulin sensitivity	(Udayappan et al., 2016; Cuevas-Tena et al., 2018)

Direction of arrows denotes to an increase (↑) or decrease (↓) relative to the discussed element.

## 4 Conclusions and future perspectives

In this comprehensive exploration of legume phytochemicals and their significant role in shaping the functional diversity of soil and gut microbes, we have elaborated on intricate functional interplay between plants, environment, plus human and farmed-animal health. These concepts are crucial not only for the One Health approach but also for achieving the goals of the European ‘Soil Mission’ (EuropeanComission, 2023). Legume phytochemicals, including saponins, flavonoids, other polyphenols, and RFOs, have a multifaceted impact. Notably, the impact of legume phytochemicals extends beyond the edible portion of the plant. Flavonoids released by legume roots function as signalling molecules, initiating a molecular dialogue that results in the formation of nodules and BNF. Furthermore, phytochemicals can shape the competitive landscape of soil microbiome, favouring certain microbial groups while inhibiting others. Some phytochemicals, like organic acids involved in nutrient cycling and tannins with their metal-chelating properties, enhance overall soil health and fertility. However, there are also examples of phytochemicals, such as alkaloids, that have the potential to negatively impact plant-associated microbiota.

From a dietary standpoint, legume phytochemicals can support beneficial gut bacteria like *Bifidobacterium* and *Lactobacillus*. This, in turn, leads to the production of SCFAs, such as acetate, propionate and butyrate, which contribute to maintaining gut health. In addition to their prebiotic potential, these compounds have anti-inflammatory properties and health-promoting effects that, when consumed as part of a balanced diet, can reduce the risk of various diseases, including gastrointestinal disorders.

While our understanding of the impact of legume phytochemicals on soil and gut microbes has greatly expanded, numerous challenges and avenues for future research remains. The complex dynamics of microbial communities in response to these compounds are not fully understood, and there is a need for investigations into the specific mechanisms through which legume phytochemicals influence human physiology. Additionally, there is a wealth of underutilised legume crops that have not been thoroughly explored, and which may possess richer phytochemical profiles which are crucial for tailoring agricultural and dietary strategies to influence microbial communities.

In conclusion, legume phytochemicals play a significant role in bridging the gap between agriculture, human and animal health. Moving forward, interdisciplinary collaboration among researchers, farmers, ecologists, and public health advocates will be essential to harness the full potential of these remarkable plant compounds for a sustainable and healthier future.

## Author contributions

RD: Writing – original draft, Writing – review & editing. PI: Writing – review & editing. AG: Writing – review & editing. MV: Writing – original draft, Writing – review & editing.

## Footnotes

1 Bloat is a condition which results from the complex interactions of feed qualities, rumen microbial diversity, and physiological (and genetic) aspects of the host. Factors leading to bloat include (high) soluble protein levels (in feed), digestion rates, microbial slime production in the rumen, and the passage rate of digested material (Wang, Y., Majak, W., & McAllister, T. A. (2012). Frothy bloat in ruminants: cause, occurrence, and mitigation strategies. Animal feed science and technology, 172(1-2), 103-114.
